# The Status of the EGFR T790M Mutation is associated with the Clinical Benefits of Osimertinib Treatment in Non-small Cell Lung Cancer Patients: A Meta-Analysis

**DOI:** 10.7150/jca.38411

**Published:** 2020-03-04

**Authors:** Zhe Zhao, Lu Li, Zhijie Wang, Jianchun Duan, Hua Bai, Jie Wang

**Affiliations:** 1 National Cancer Center/National Clinical Research Center for Cancer/Cancer Hospital, Chinese Academy of Medical Sciences and Peking Union Medical College, Beijing, 100021, China.; 2 State Key Laboratory of Molecular Oncology, Department of Medical Oncology, National Cancer Center/National Clinical Research Center for Cancer/Cancer Hospital, Chinese Academy of Medical Sciences and Peking Union Medical College, Beijing, 100021, China.; 3 Health Service Department of the Guard Bureau of the Joint Staff Department, Beijing, 100017, China.

**Keywords:** non-small cell lung cancer, EGFR T790M mutation, Osimertinib, resistance, meta-analysis

## Abstract

**Background and Purpose**: Pervious studies have demonstrated that the loss of EGFR T790M after Osimertinib treatment may be the cause of Osimertinib resistance. Here, we conducted a meta-analysis to evaluate the association between the persistence of EGFR T790M and the clinical benefits of Osimertinib in non-small cell lung cancer (NSCLC) patients with baseline EGFR T790M mutation.

**Experimental design and Methods**: PUBMED, EMBASE, and Cochrane databases were searched for eligible studies that provided the survival outcomes including overall survival (OS), progression-free survival (PFS) or time to discontinuation (TTD) data for each patient treated with Osimertinib with the status of the T790M mutation tested after Osimertinib resistance. The hazard ratios (HRs) and their 95% confidence intervals (CI) were calculated for each study.

**Results**: In total, eight eligible studies were included in the analysis, among which six studies provided the data on PFS, and the other two studies provided the TTD data. Overall, 312 patients (151 patients with the persistence of T790M) were identified. The persistence of T790M was associated with longer PFS (HR, 0.40; 95% CI, 0.19-0.84; *P*=0.01) and TTD (HR, 0.54; 95% CI, 0.39-0.76; *P*=0.0004). Furthermore, overall analysis the survival outcomes including PFS and TTD subgroups also showed preferable clinical benefits for patients with the T790M persistence (HR, 0.57; 95%CI, 0.45-0.73; *P*<0.00001).

**Conclusions**: Our findings confirm the persistence of T790M is associated with the clinical benefits of Osimertinib in NSCLC patients with baseline EGFR T790M mutation treated with Osimertinib.

## Introduction

Lung cancer is the most common cancer type and is one of the major causes of cancer-related deaths worldwide 1. The clinical management of lung cancer has in recent years taken advantage of the increasing awareness of pathogenesis and drug resistance at the molecular level of tumors, especially in non-small cell lung cancer (NSCLC). Epidermal growth factor receptor (EGFR) gene mutations are common drivers of NSCLC. EGFR tyrosine kinase inhibitors (EGFR-TKIs) have revolutionized the treatment for NSCLC patients with EGFR driver mutations 2, 3. However, although these new generations of TKI-targeted drugs have higher disease control rates and initial response rates in NSCLC patients with EGFR-sensitive mutations, the emergence of acquired resistance to EGFR-TKIs is inevitable. Tyrosine790 is a “gatekeeper” residue that is integral for regulating the affinity of TKI-targeted drugs in the ATP binding pocket, while about 50-60 % of patients have a secondary T790M mutation. With the Tyrosine790 mutation, the ATP binding pocket has increased affinity for ATP and confers resistance due to competition with EGFR-TKIs 4.

The EGFR T790M mutation is specifically targeted by the third-generation EGFR-TKI Osimertinib. For patients with the T790M mutation, Osimertinib has provided significant survival benefits compared to cytotoxic chemotherapy and has been approved as a standard treatment 5-7. In the AURA3 study, the median PFS was significantly prolonged in patients receiving Osimertinib (10.1 vs. 4.4 months; *P*<0.001). The objective response rate of Osimertinib was significantly higher compared with chemotherapy (71% vs. 31%; *P*<0.001) 8. In addition, Osimertinib was used as a first-line therapy for NSCLC patients with EGFR-mutations, and median PFS was significantly longer than first-line EGFR-TKIs (18.9 and 10.2 months, respectively; *P*<0.0001) in the FLAURA study 9.

Unfortunately, even in T790M-positive tumors, the response to Osimertinib is not permanent and drug resistance will occur sooner or later. Complex mechanisms that mediate resistance to Osimertinib have been demonstrated, such as the acquisition of tertiary EGFR mutations (e.g. EGFR 797S, L718Q mutations), HER2 or MET amplification, BRAF mutations, and the histologic changes to small cell transformation 10. It is worth noting that even though the mechanisms of Osimertinib resistance are heterogeneous, they can be conceptualized as binary variables: some patients lose secondary T790M mutations when they acquire Osimertinib resistance, while some patients remain a T790M positive status 11. Previous reports have shown that changes in T790M mutation status appear to be associated with the clinical benefits of Osimertinib in baseline EGFR T790M-positive NSCLC patients 12-14. A study by Lin et al. consistently found that loss of the T790 M mutation was associated with the shortest PFS (median 2.6 months, 95% CI 1.3-NR) of the Osimertinib resistance group 12. Similarly, a retrospective study conducted by Zhou et al (2018) also demonstrated a shorter PFS in patients with T790M loss when patients acquired Osimertinib resistance 13. Overall, studies have found that the presence or absence of the T790M mutation may be associated with resistance to Osimertinib, but the mechanism of this action remains unknown 15-17. Hence, we conducted a meta-analysis of the published literatures to explore the association between the persistence of T790M and the clinical benefits of Osimertinib in NSCLC patients with baseline EGFR T790M mutation.

## Methods

### Search strategy

This study was conducted in compliance with the recommendations of the Cochrane Handbook for Systematic Reviews of Interventions and reported based on the Preferred Reporting Items for Systematic Reviews and Meta-Analyses (PRISMA) statement guidelines 18. PUBMED, EMBASE, and Cochrane databases were searched for eligible published articles that reported survival time for each Osimertinib treated patient with both positive baseline EGFR T790M and EGFR T790M detection after Osimertinib resistance nearly 5 years from January 1^st^, 2014 to September 1^st^, 2019. The search terms utilized included: non-small cell lung cancer, T790M, epidermal growth factor receptor, and Osimertinib. Case reports, letters, conference abstracts, comments, editorials, proceedings, and personal communications were excluded. Moreover, the reference lists of all trials fulfilling the eligibility criteria were examined for any relevant studies missed in initial searches.

### Data extraction and quality assessment

The following information was extracted from the chosen studies: first author or correspondent author's name, publication year, study design, baseline characteristics, number of participants and major outcomes of T790M persistence and loss when Osimertinib progressed, and survival outcome for each patient. Two researchers independently extracted the data, and any discrepancy was resolved by discussion. The quality of the included studies was assessed using the Newcastle-Ottawa Scale (NOS) independently by two researchers 19.

### Statistical analysis

For each individual study, HR with a survival result of 95% CI was extracted. If there was no HR of 95% CI, the hazard ratios and corresponding 95% confident intervals for each included study were calculated using the Cox regression model. The method of fixed or random-effects inverse-variance- weighted was then used to pool the hazard ratios. A χ2-based homogeneity test was performed and the inconsistency index (I^2^) and Q statistics were determined. We classified the I^2^ value<50% as having homogeneity and a fixed-effect model was well accepted. An I^2^ value>50% predicted potential heterogeneity. If heterogeneity existed, subgroup analysis was used to weaken its effects. Else, we synthesized the results with a randomized effect model if no definite heterogeneity was detected. Pooling effects were calculated and a two-sided p-value<0.05 was considered to indicate statistical significance. Sensitivity analysis was performed using the leave-one method. At last, the publication bias was analyzed. All analyses were performed using the comprehensive Meta-analysis statistical software Review manager 5.2 (The Nordic Cochrane Centre, Rigshospitalet 2008).

## Results

### Selection of eligible studies

**Figure 1:**
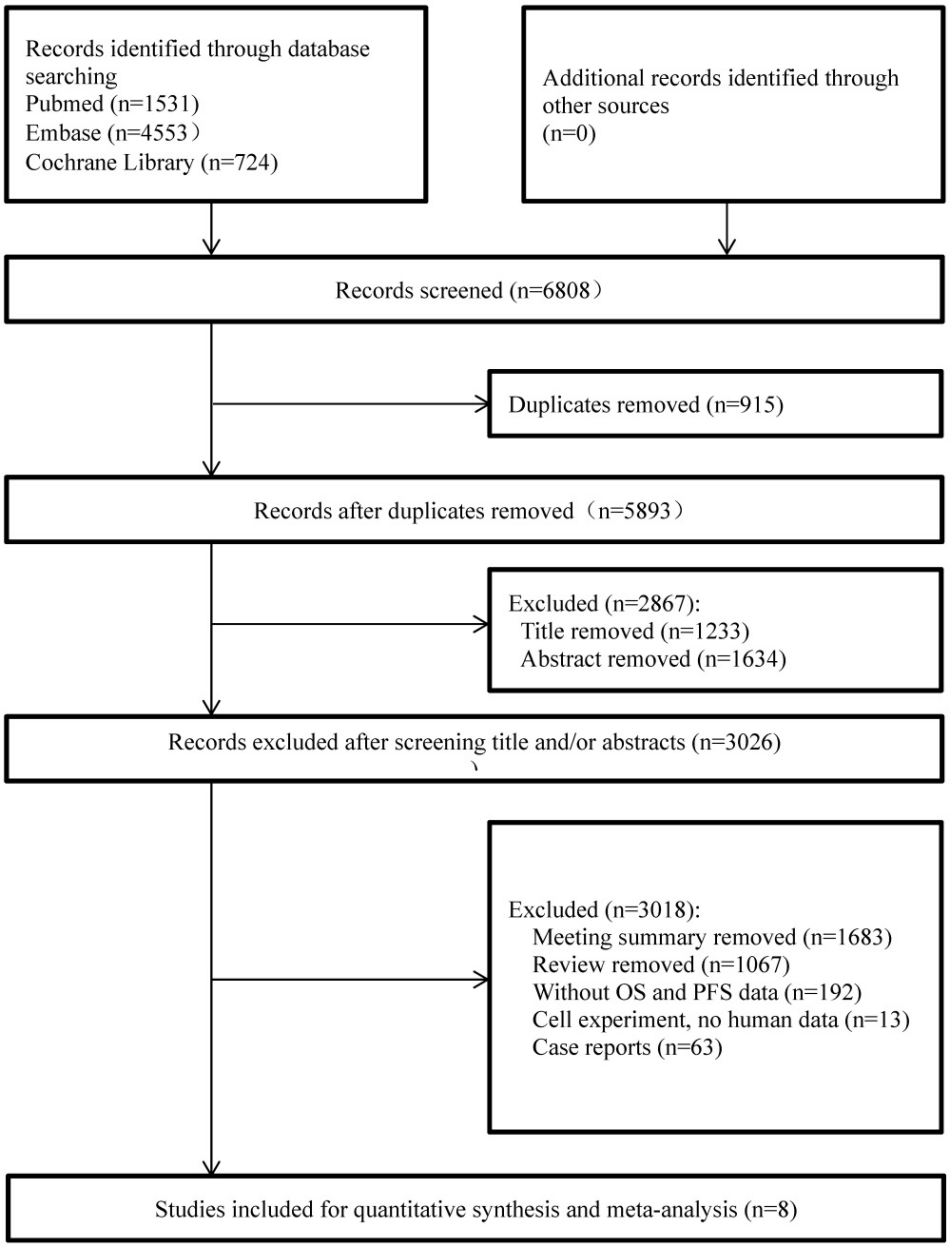
Study flow diagram.

The initial search strategy identified a total of 6,808 related articles, of which 1,531 were from PUBMED, 4,553 were from EMBASE, and 724 were from the Cochrane Library. Due to duplications, we deleted 915 studies. After the titles and abstracts were screened, 2,867 studies were excluded because they did not meet the inclusion criteria. Then, we carefully reviewed the full text of the remaining 3,026 eligible studies and excluded 1,683 conference abstracts, 1,067 reviews, 63 case reports, 192 articles without specific PFS or TTD data, and 13 studies related to cell experiments. After filtering, a total of eight clinical studies were selected for final analysis. A flow chart describing the study selection eligibility is shown in Figure 1.

### Characteristics of included studies and quality assessment

A total of eight studies with 312 patients were included in the analysis. The characteristics of the included studies are outlined in Table 1. These studies included 312 patients with baseline T790M mutations who were orally treated with Osimertinib to resistance and were re-biopsied for T790M mutation status in the tissue or blood, of which outcomes included PFS and TTD, respectively. A total of 6 studies reported the outcome of PFS and 2 studies reported TTD. The NOS scores of the eight studies are listed in Table 2. In general, the studies are of good quality with the NOS scores ranging from 6 to 8 points.

### Meta-analysis

**Figure 2:**
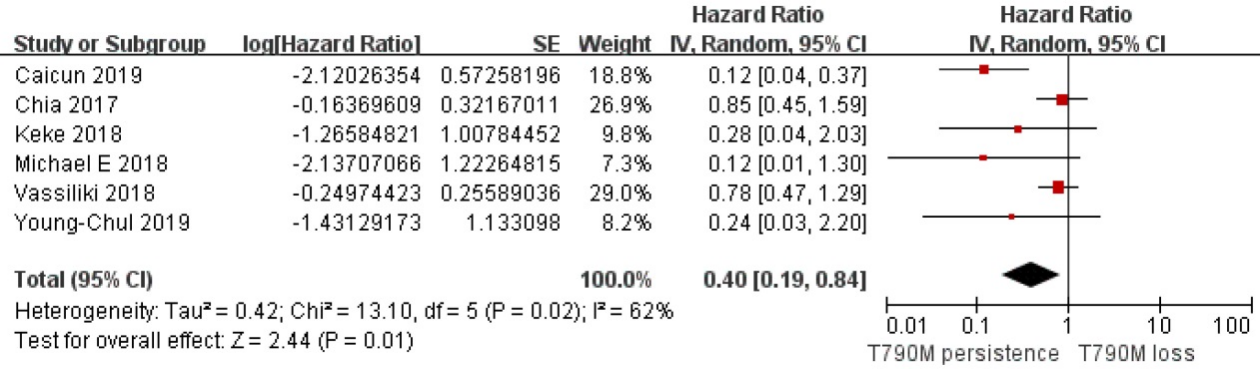
Forest plot of PFS included in the study in the persistence of T790M NSCLC patients compared with the T790M loss group when they acquired resistance to Osimertinib and had a positive T790M mutation at baseline.

**Figure 3:**
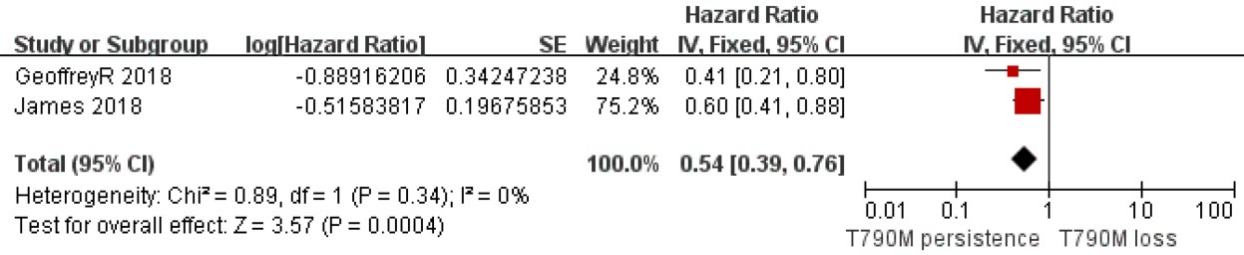
Forest plot of TTD included in the study in the persistence of T790M NSCLC patients compared with the T790M loss group when they acquired resistance to Osimertinib and had a positive T790M mutation at baseline.

**Figure 4:**
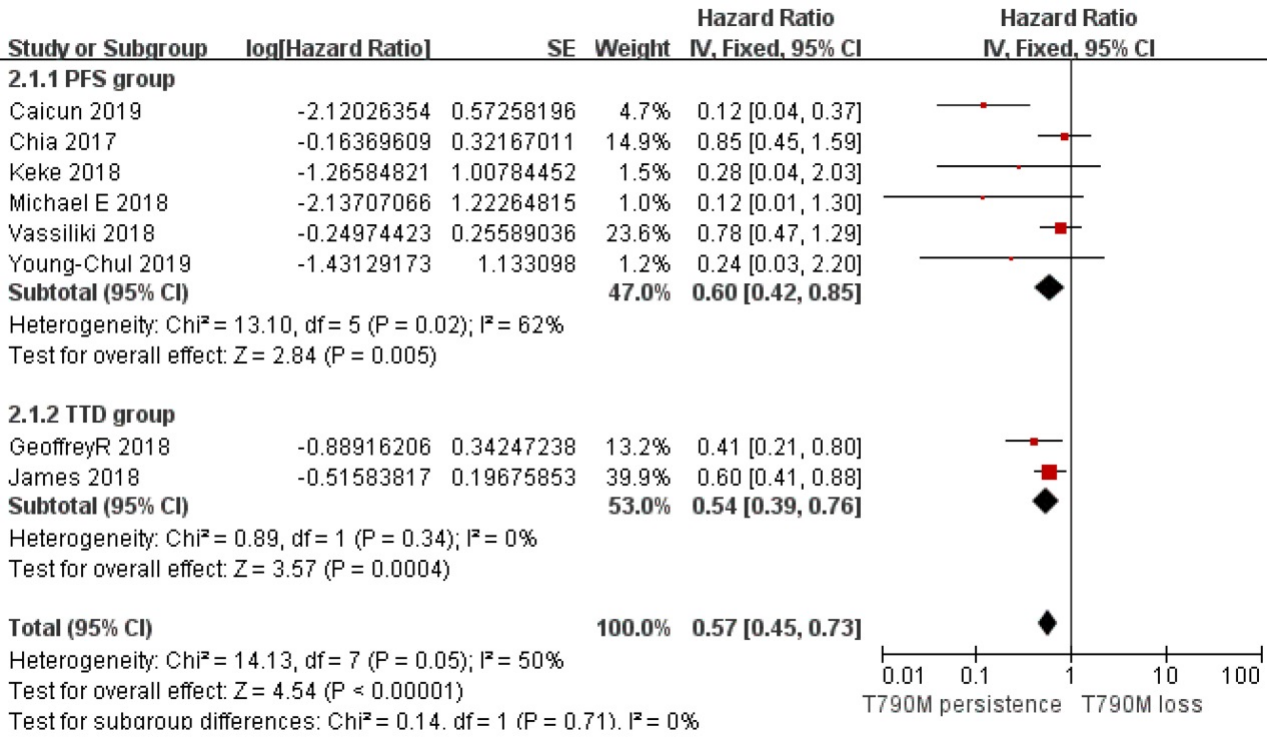
Forest plot of survival outcomes included in the study in the persistence of T790M NSCLC patients compared with the T790M loss group when they acquired resistance to Osimertinib and had a positive T790M mutation at baseline.

Of the 312 patients, 151 had T790M mutation persistence after Osimertinib resistance, while 161 lost the T790M mutation with resistance to Osimertinib. Analysis of the PFS data provided by 6 clinical studies showed that the T790M persistence group had a longer PFS than the T790M loss group (pooled HR=0.40; 95% CI 0.19 to 0.84; *P*=0.01; I^2^=62%; Figure 2). Considering the very nature of the retrospective design, there was potential heterogeneity among included studies. We classified I^2^ higher than 50% which is acceptable and does not affect the reliability of the results. In addition, an analysis of the other two studies on TTD also supported a longer TTD in the T790M persistence group vs. the T790M loss group (pooled HR=0.54; 95% CI 0.39 to 0.76; *P*=0.0004; Figure 3) without heterogeneity (I^2^=0). Although only two studies provided the TTD data, the study showed that the heterogeneity between them was zero, which supported the result was credible. Considering that both PFS and TTD belonged to the survival outcomes, we comprehensively analyzed the survival outcomes of the two subgroups, which showed that the T790M persistence group had longer survival outcomes than the T790M loss group (HR=0.57; 95% CI 0.45 to 0.73; *P*<0.00001; Figure 4), whose I^2^ equaled 50% due to the potentially acceptable heterogeneity from the included retrospective study. Only one study showed specific OS data, so we did not include it in our meta-analysis. However, the study also showed that the OS data of the T790M loss group was also decreased than that of the persistence group (*P*=0.021)^13^. From this we believe that the persistence of T790M is associated with the benefits of clinical outcomes in NSCLC patients treated with Osimertinib.

### Sensitivity analysis

Sensitivity analysis assesses the stability and reliability of the results by omitting one study at a time. The results showed that the combined effect size and heterogeneity analysis in Review Manager 5.2 were not significantly affected by individual studies, indicating that the meta-analysis results were stable and reliable.

### Publication bias

Due to the small number of studies involving PFS and TTD, the publication bias was not assessed with the Egger test. However, given the objective existence of publication bias in the retrospective study and the overall heterogeneity between them was favorable, we believe that publication bias would not affect the reliability of our results.

## Discussion

In the study, we demonstrated that the presence of T790M after Osimertinib resistance was associated with longer survival benefits of Osimertinib in T790M positive NSCLC patients treated with Osimeritinb. These results suggest that the detection of T790M after treatment may serve as a potential predictor for the benefit of Osimertinib in patients with NSCLC.

Although more and more studies have confirmed the superior efficacy of Osimertinib in NSCLC paitents with EGFR driver mutations 5, 12, 20-22, the tumor response and survival outcomes after the treatment of Osimertinib are usually different in patients. The mechanisms of resistance to Osimertinib have been the subject of several clinical studies 23-25. Previously prospective or retrospective clinical studies have compared the relationship between treatment outcomes and T790M mutation status in disease progression, but without a definitive conclusion. Our meta-analysis focused on the NSCLC patients with baseline T790M mutation who had re-tested for T790M mutations in disease progression after single-agent Osimertinib treatment. We found that the T790M mutation could not be detected in 51.6% (161/312) of the patients when they were resistant to Osimertinib. Importantly, the loss of the T790M mutation was significantly associated with poor survival. Conversely, the persistence of T790M-positive mutations in clinical progression of oral Osimertinib appeared to be a good predictor of treatment outcome.

**Figure 5:**
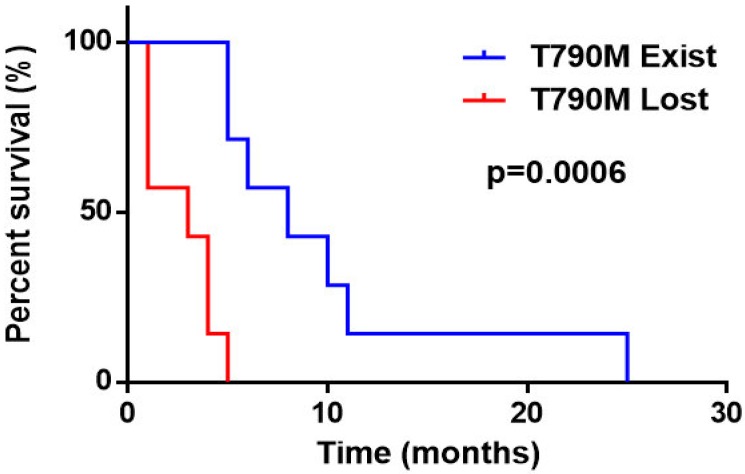
Progression free survival of T790M persistence vs. T790M loss after Osimertinib resistance in NSCLC patients with the EGFR T790M mutation at baseline in our hospital.

What's more, in order to verify our conclusions, we further reviewed the clinical information of 214 NSCLC patients with the EGFR T790M mutation at baseline treated with Osimertinib in our hospital. Of these patients, 38 patients were excluded from the analysis due to irregularly taking Osimertinib. 79 patients had neither disease progression nor resistance and 3 patients discontinued Osimertinib because of adverse reactions. The remaining 94 patients with the EGFR T790M mutation at baseline suffered from disease progression after Osimertinib treatment. While, tissue or blood genetic detections were re-executed on 14 of the 94 patients with complete clinical data after resistance to Osimertinib. The genetic status of these 14 patients and their clinical progression-free survival data were used to validate our conclusions. Of the 14 patients, 7 had T790M mutation persistence after Osimertinib resistance, while 7 lost the T790M mutation with resistance to Osimertinib. Analysis of the PFS data showed that the T790M persistence group had a longer median PFS than the T790M loss group (8 months vs. 3 months, respectively; pooled HR=0.098; 95% CI 0.019 to 0.513; P=0.0006) (Figure 5), which validated our main findings and conclusions.

In addition, the mechanism of the difference in T790M mutation status when patients acquired resistance to Osimertinib remains unclear. The most likely explanation may be the genomic heterogeneity inherent in the tumor before the treatment with Osimertinib 26, 27. As previously reported, concomitant genomic alterations are widespread in lung cancer 28, and the T790M-positive and wild-type cell clones may co-exist in tumors at baseline levels or after acquired resistance to pre-EGFR TKI 29. Osimertinib is effective for patients who have EGFR T790M mutations and can exert selective pressure, resulting in an increase in pre-existing T790M wild-type clones with additional EGFR-independent resistance mechanisms. This makes them more visible than T790M mutant cells in primary “T790M positive tumors”, which may further result in the loss of T790M under resistance to Osimertinib 30. Consistent with this hypothesis, we statistically analyzed the results of second-generation sequencing of tumors that have lost T790M mutations and found multiple EGFR-independent resistance mechanisms, such as alternative signaling pathways for bypassing activation and histological transformation 31, 32. We included a summary of 14 studies with Osimertinib resistance mechanisms and summarized 134 patients who had T790M loss after Osimertinib resistance and re-tested for blood or tissue. The results showed the occurrence of the following mutations: 18.7% (25/134) had TP53 mutations; 17.8% (24/134) had MET mutations; 11.2% (15/134) had small cell lung cancer histological pathology transformation; 5.6% (8/134) had the C797S mutation; 5.6% (8/134) had the KRAS mutation; 5.6% (8/134) had the PIK3CA mutation; 3.7% (5/134) had the BRAF mutation; 2.8% (4/134) had the CCNE1 amplification; 2.8% (4/134) had the CDK6 amplification; 2.8% (4/134) had the ratio of CCDC6-RET fusion; and the ratio of HER2 amplification occurred in 1.9% (3/134). Besides, there were many other mutations that were difficult to count one by one, but it had to be considered that the occurrence of these downstream mutations may provide a potential mechanism for Osimertinib resistance after the T790M mutation disappears. What's more, due to the lack of T790M mutations, there may be EGFR-independent resistance mechanisms, which need further study.

Previous studies have shown that the dynamic quantitative assessment of the T790M mutation load is related to the extent of response to third-generation EGFR inhibitors 13-15, 33-36. Our results provide further evidence that the current binary assessment (presence or loss) of T790M status alone may not be a uniform biomarker for Osimertinib treatment, but the detailed analysis of tumor genomic changes before and after treatment highlight the role of the development of Osimertinib resistance. The results of this study may provide a new understanding of the mechanisms that drive early Osimertinib resistance. Overall, an understanding of the detailed genomic alterations of tumors before and after the Osimertinib treatment is not yet fully understood. More clinical data are needed to reveal the difference in T790M mutation status after Osimertinib treatment failure.

Our study has several limitations. First, for the studies were retrospective in nature, and there might have been potential selection bias. Second, the sample size of included studies was relatively small. Third, because of the limited numbers of studies and the types of publications included, publication bias was not assessed with the Egger test.

In summary, our meta-analysis indicates that persistence of the T790M mutation is associated with longer survival benefits of the use of Osimertinib in non-small cell lung cancer with a T790M mutation at baseline. Dynamic detection of T790M mutation status may help to indicate and predict disease progression in a timely manner.

## Figures and Tables

**Table 1:** Characteristics of included studies.

Studies	Type	Year	Nation	Age	Gender	Re- biopsies	T790M positive	T790M negative	Outcome	Pos/Neg HR	95% CIs
Caicun 2019 13	retrospective	2019	China	<65(20) >=65(11)	F(16), M(15)	31	16	15	PFS	0.12	0.039-0.368
GeoffreyR 2018 14	retrospective	2018	American	Unknown	F(28), M(13)	41	14	27	TTD	0.411	0.21-0.804
James 2018 34	retrospective	2017	American	Unknown	uncategorized	110	58	52	TTD	0.597	0.406-0.878
Vassiliki 2018 35	prospective	2018	UK	Unknown	uncategorized	64	28	36	PFS	0.779	0.472-1.287
Michael E 2018 36	retrospective	2018	American	59	F(5), M(4)	9	7	2	PFS	0.118	0.011-1.327
Keke 2018 10	retrospective	2018	China	66	F(5), M(4)	9	7	2	PFS	0.282	0.039-2.027
Chia 2017 12	prospective	2017	Taiwan	59	uncategorized	41	18	23	PFS	0.849	0.452-1.595
Young-Chul 2019 15	prospective	2019	Korea	Unknown	uncategorized	7	3	4	PFS	0.24	0.03-2.20

**Table 2:** Quality assessment included in the study - using the NOS score.

Studies	Selection 1	Selection 2	Selection 3	Selection 4	Comparability	Outcome assessment			Score
						1	2	3	
Caicun 2019^13^	*	*	*	*	*	*	*	*	********
GeoffreyR 2018^14^	*	*	*	*	*	*	*	*	********
James 2018^34^	*	*	*	*		*	*	*	*******
Vassiliki 2018^35^	*	*	*	*	*	*	*	*	********
Michael E 2018^36^	*		*	*	*	*	*	*	*******
Keke 2018^10^	*		*	*		*	*	*	******
Chia 2017^12^	*	*	*		**	*	*	*	********
Young-Chul 2019^15^	*		*	*	*	*	*	*	*******

Notes In this evaluation form, *represents one point, ** represents two points, ******or more indicates that the quality of the article is high and credible out of 9 points.

## Abbreviations

EGFR: epidermal growth factor receptor
EGFR- TKI: epidermal growth factor receptor-tyrosine kinase inhibitor
OS: overall survival
PFS: progression-free survival
TTD: time to discontinuation
HR: hazard ratio
NSCLC: non-small cell lung cancer
ATP: adenosine triphosphate
